# Association of problematic Internet use and oral health-related quality of life among medical and dental students

**DOI:** 10.1186/s12909-021-03092-x

**Published:** 2022-01-03

**Authors:** Halimeh Ghareghol, Mina Pakkhesal, Aliakbar Naghavialhosseini, Amir reza Ahmadinia, Nasser Behnampour

**Affiliations:** 1 School of Dentistry, Golestan University of Medical Sciences, Gorgan, Iran; 2 Dental Research Center, Community Oral Health Department, School of Dentistry, Golestan University of Medical Sciences, Gorgan, Iran; 3 Dental Research Center, Orthodontics Department, School of Dentistry, Golestan University of Medical Sciences, Gorgan, Iran; 4 Dental Research Center, Periodontics Department, School of Dentistry, Golestan University of Medical Sciences, Gorgan, Iran; 5 Department of Biostatistics and Epidemiology, School of Health, Golestan University of Medical Sciences, Gorgan, Iran

**Keywords:** Internet use, Oral health, Quality of life, Medical students, Dental student

## Abstract

**Background:**

The Internet as a communication tool is an essential component of daily life. Nowadays, problematic Internet use (PIU) has led to various psychosocial problems that can indirectly lead to oral diseases due to neglect of healthy behaviors. Also, college students are a large proportion of Internet users. The present study aimed to determine the association between problematic Internet use and oral health-related quality of life (OHRQoL) among medical and dental students.

**Methods:**

A cross-sectional descriptive-analytical study was conducted on medical and dental students in the first and second years of education (basic sciences courses) at the Golestan University of Medical Sciences, Iran, between January and July 2020. The data collection process was carried out in the following sequence: questionnaire on demographic characteristics (age, gender, marital status, academic field, and year); Problematic Internet Use Questionnaire (PIUQ); and Oral Health Impact Profile (OHIP-14) questionnaire.

**Results:**

Among 268 medical and dental students, 171 students (63.81%) [95% confidence interval: 58.02%- 69.60%] had problematic Internet use. The mean PIU score in the first-year was significantly higher than the second-year students. In addition, 65% of single students and 25% of married subjects were dealing with PIU. The statistical difference between mean OHIP scores among PIU students (12.5 ± 2.9), with average Internet usage (7.39 ± 6.6), was significant. The Spearman correlation coefficient between PIU and OHIP was 0.309 and significant (P-value < 0.000001). It indicates that students with higher PIU showed higher OHIP scores.

**Conclusion:**

The present study showed that problematic Internet use was significantly associated with oral health-related quality of life (OHRQoL) among first and second-year medical and dental students. Thus, the students with problematic Internet use experienced a poorer oral health-related quality of life than average Internet users. Furthermore, appropriate preventive and interventional strategies need to be developed to encourage rational use of the Internet to protect the users' oral health, especially among medical and dental students.

## Background

The internet use has increased very quickly around the world in the recent past. The most important challenge that we face with internet is the internet addiction that is associated with loss of control of the user. Internet behaviors have been described as “problematic”, “excessive”, “addiction”, “dependence”, “pathological”, “impulsive”, “compulsive”, or “abnormal”, or prefixed as “hyper-” to present disease states [1]. Nowadays, the Internet has developed fundamentally, and the technology has advanced medical education and practice worldwide. Medical students have the ability to keep themselves up to date with the exponential growth knowledge to become lifelong learners. The Internet has provided better opportunities for gathering information, social interaction, and entertainment in digital life. Moreover, medical schools in both developed and developing countries utilize educational technology to bring effective medical education changes [2]. College students may be especially vulnerable to developing problematic Internet use for reasons that include free Internet access, courses that require its use, and the sudden freedom from parental control and monitoring, especially because many students live in dormitories [3]. Young university students are in a dynamic transition period of growth and development that bridges adolescence (high school students) and adulthood (people in the community). Since many of them are living away from home for the first time in their lives, their health, lifestyle, and behaviors could be easily changed [4]. In other words, students with a lower university year are more vulnerable to having problematic Internet use. The young students experience freedom and relief from the parental control for the first time in their lives [5]. Prior research among adolescents and youths, including college students, has also found that the prevalence of internet addiction in this group ranged from 5.9% to 26.8% [6]. In addition, health professional students, such as medical and dental students, are susceptible groups based on their time on the Internet [1]. It is notable that the problematic Internet use is associated with an unhealthy lifestyle such as inactive behaviors, eating disorders, unusual meals, unhealthy eating habits, later bedtimes, and less sleep [7–10]. Moreover, excessive computer use may be associated with adverse oral health behaviors and poor oral health because it displaces time spent in personal hygiene care and medical care. So, adolescents with problematic Internet use could skip meals, have an unbalanced diet, and have insufficient sleep, all of which might be associated with poor oral health [11]. Oral health is described as a “convenient and functional system which allows people to continue their social role”. Also, oral health is essential to general health and wellbeing. Therefore, oral health is more than the absence of dental caries or periodontal disease, or even having healthy teeth [12]. Although oral health problems influence eating habits, talking, and social life, they might reduce people’s quality of life [13–15]. The concept of Oral Health-related quality of life (OHRQoL) uses patient-centered outcome measures to identify the impact of oral health on aspects of everyday life in terms of a person’s functional, social, and psychological well-being [16].

The present study’s aims were to determine problematic Internet use prevalence and its association with oral health-related quality of life among medical and dental students studying basic sciences at Golestan University of Medical Sciences, Gorgan, Iran.

## Methods

A cross-sectional descriptive-analytical study was conducted on medical and dental students (first & second-year) of the Golestan University of Medical Sciences in Gorgan, north-east of Iran, between January and July 2020. The study began after being approved by the local Research Ethics Committee (IR.GOUMS.REC.1398.082) and was performed according to the Declaration of Helsinki. All participants signed written informed consent forms before taking part in the study. The present study was administered at the medical faculty as dental students in Gorgon city pass their basic sciences courses in the medical school departments. After passing the basic science courses in the first and second academic years, dental students in dental school and medical students in medical school continue their education. Also, similar studies have shown that students in the first or second professional years were more likely to experience problematic Internet use than higher years’ students [5, 17, 18]. This issue can be due to complicated higher academic year courses than the first and second-year courses. So, the higher years’ students probably have less free time to surf the Internet. Furthermore, special attention should be paid to earlier preventive strategies at the lower years of university students, and those should be guided to the proper use of the Internet.

Following, the investigator delivered the questionnaires to those students willing to participate in the study. The students completed the questionnaires in a classroom at the end of their classes under the supervision of the researcher (H.G.), who was present to answer any questions that might arise. Explanations were given regarding the objectives of the study and then the data collection process was carried out in the following sequence: questionnaire on demographic characteristics (age, gender, marital status, academic field, and year); Problematic Internet Use Questionnaire (PIUQ); and Oral Health Impact Profile (OHIP-14) questionnaire. Consequently, the questionnaires were collected immediately after completion. The data were handled anonymously and confidentiality in all stages of the study.

Problematic Internet Use Questionnaire (PIUQ) includes 18 items in 3 subscales of obsession, neglect, and control disorders with 5- point Likert scale answers (1 = never, 2 = rarely, 3 = sometimes, 4 = often, and 5 = very often) developed by Demetrovics [19]. The total scores of the questionnaire ranged from 18 to 90. Mazhari and colleagues validated this questionnaire among Iranian medical students [20]. According to the questionnaire's developers' suggestion, the score of 41 was determined as the cut-off point. Therefore, those with scores ≤ 41 were considered as "average Internet users", and those with scores > 41 were considered as "problematic Internet users".

The short version of the Oral Health Impact Profile (OHIP-14) questionnaire was used for assessing the OHRQoL [21]. This questionnaire was previously been validated for Iranian population [22]. OHIP- 14 questionnaire consists of 14 items in seven domains (functional limitation, physical pain, psychological discomfort, physical disability, psychological disability, social disability, and social handicap). Students were asked to indicate how frequently they experienced each problem within a reference period of 12 months on a 5-point Likert-type scale (0 = never, 1 = rarely, 2 = sometimes, 3 = often, and 4 = very often). The sum of each item's scores gives the overall score ranging from 0 to 56 points, with higher scores representing greater specific oral health problems and impacting the quality of life.

The sample size of the study was calculated based on a finite population formula. The target population in the present study was 465 Medical and Dental students that were educated in first and second academic years at Golestan University of Medical Sciences. So, the sample size was estimated as 279 students by considering a 0.03 Type I error rate, 95% confidence interval, 10% possibility of sample loss, and 21% prevalence rate of PIU reported in previous study [20]. Quota sampling was employed to ensure the representativeness of the medical and dental students’ numbers diversity within the university. Also, the survey was conducted based on the input of volunteer students until the desired number is reached in each quota. In the current study all students who had inclusion criteria have an equal chance of getting included in the study. Finally, among 279 questionnaires, 11 forms were excluded from analysis due to multiple missing entries. Thus, the response rate was 96%, and 268 valid surveys were included in the final analyses.

The collected data were analyzed using the Statistical Package for the Social Sciences (SPSS, version 18.0). The Shapiro–Wilk test determined the normality of the data. A Levene’s test was used to check the homogeneity of variances. T-test was used to compare means between two independent groups with a normal distribution, and the Mann–Whitney test was used for data that did not have a normal distribution. P-values less than 0.05 were considered significant.

The association between PIU and OHIP was determined in two methods: classification on the presence of problematic Internet use (using a cut-off score of 40 on the PIU score), and correlation coefficient (Spearman’s rho).

## Results

In this study, the mean age of participants was 20.85 ± 2.66 years. Among 268 students, 94 respondents were dental students, and 174 persons were medical students. The male students made up 48.9% (n = 131), while the female students made up 51.1% (n = 137). Most of them (97%) were single, and 55% were studying in the first and 44% in the second year. Also, 139 students (51.9%) were from a public university, and 129 subjects (48.1%) were from a private university.

The prevalence of problematic Internet use among medical and dental students in basic science period was 63.81% (95% confidence interval: 58.02- 69.60). The results showed that mean of PIU scores was significantly higher in the first-year (48.81) than in the second-year (43.12) students (P-value = 0.002). Besides, the relationship between age and the mean of PIU scores was reverse and statistically significant according to the Spearman correlation coefficient (r =—0.225, P-value = 0.0002). There was no significant difference in the mean of PIU scores between other studied variables. Furthermore, mean of OHIP scores was significantly higher in the male (12.33) than in the female (9.02) students (P-value = 0.002).

As shown in Table 1, according to classification on the presence of problematic Internet use, the mean of OHIP scores was significantly higher in problematic Internet users than average Internet users (P-value = 0.0001). Also, it was significant in all of 7 OHIP domains (P-value < 0.05).

**Table 1:** Mean comparison of OHIP scores in 7 domains between average and problematic internet users among medical and dental students (*n* = 268)

		**Problematic Internet use** **(*n* = 171)**	**Average Internet use (*n* = 97)**	***P*-value**^**a**^
**Total OHIP scores (Mean ± SD)**		12.50 ± 9.26	7.39 ± 6.64	0.0001
**OHIP domains**	Functional limitation	1.57 ± 1.49	0.74 ± 1.05	0.0001
	Physical discomfort	2.24 ± 1.63	1.57 ± 1.39	0.001
	Psychological discomfort	2.41 ± 1.78	1.83 ± 1.49	0.011
	Physical disability	1.58 ± 1.70	0.71 ± 1.00	0.0001
	Psychological disability	1.85 ± 1.63	1.21 ± 1.40	0.001
	Social disability	1.40 ± 1.59	0.64 ± 1.12	0.0001
	Social handicap	1.42 ± 1.64	0.65 ± 1.33	0.0001

^a^Associated to the Mann–Whitney test

**Fig. 1:**
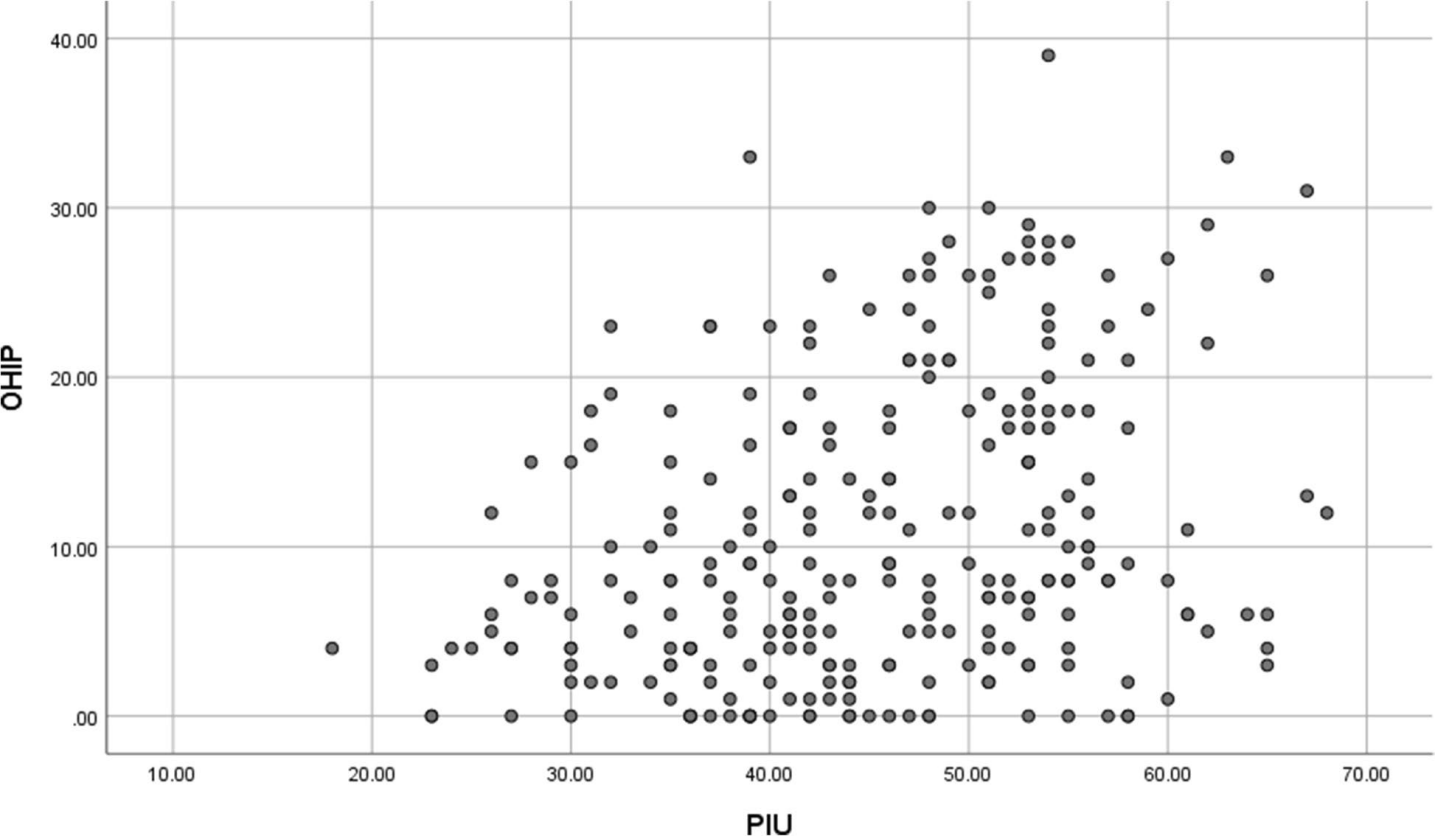
Association between problematic internet use (PIU) and oral health impact profile (OHIP) among the responders (*n* = 268); Spearman correlation coefficient = 0.309

Also, the Spearman correlation coefficient between PIU and OHIP was 0.309 and significant (P-value < 0.000001). Too, the positive association between PIU and OHIP scores was illustrated in Fig. 1. As observed, high PIU scores had concomitance with high OHIP scores.

Then, Spearman correlation coefficient was used to adjust studied variables such as gender, academic year, and marital status (Table 2). The results indicated that association between PIU and OHIP in female students was higher and stronger than male students. Also, the mentioned association in second year students was higher and significant than first year students. However, the association between PIU and OHIP in married students was higher than single students; but it was not significant.

**Table 2:** Spearman correlation coefficient between problematic internet use (PIU) and oral health impact profile (OHIP) among different subgroups of medical and dental students (*n* = 268)

**Variables**	**correlation coefficient**	***P***-value^**a**^
**Gender**		
Male	0.247	0.004
Female	0.362	0.000015
**Academic year**		
First year	0.260	0.001
Second year	0.327	0.0003
**Marital status**		
Single	0.306	< 0.000001
Married	0.370	0.367

^a^Associated to the Spearman’s rho

## Discussion

The present data indicates that more than half of the first and second year medical and dental students have problematic Internet use. Also, the evaluated association between problematic Internet use and oral health-related quality of life indicates that PIU had a direct relation with OHRQoL (P < 0.05). It seems that the students with problematic Internet use experienced a poorer oral health-related quality of life than average Internet users.

On the other hand, the length of the general dentistry curriculum in the educational system of Iran is 6 years or 12 semesters, in which the courses are presented in two stages. In the first stage, dental students take basic sciences courses (such as anatomy, immunology, physiology, biochemistry, microbiology, pathology, and pharmacology) during the first and second academic years. At the end of the first 2 years, students take a comprehensive examination in the basic sciences. After successfully passing the courses in the first stage and the comprehensive examination, students take the preclinical and clinical courses in the second stage, which lasts for the remaining 4 years, or 8 semesters. Also, the conventional medical curriculum consists of 15 semesters in Iran. Medical students should pass four main stages: basic sciences, physiopathology, externship, and internship.

Internet addiction is recognized as a worldwide health problem [23]. Hence, several studies have shown that problematic Internet use could lead to mental health complications such as stress and depression, which can eventually result in poor oral hygiene [7, 24–26]. Also, a negative association was revealed between problematic Internet use and frequent tooth-brushing and a direct association between problematic Internet use and poor oral health and oral symptoms [27]. Al-Ansari et al.’s study showed that, among participants with good and fair perception of oral health, average and frequent Internet users had less negative oral health practices (sugar and tobacco consumption) and more positive oral health practices (oral hygiene) than participants with problematic Internet use [28].

Despite the growing interest of researchers in assessing the Internet overuse effects, heterogeneity in the studied population and using different measurement tools make it difficult to interpret and compare current research findings with other similar studies. Notably, no similar study examined both the OHRQoL and the problematic Internet use. One research that was relatively the same as the present study was conducted on middle and high school students [3], and showed the association between signs and symptoms of oral disease in high-risk Internet use. Furthermore, comparing the related studies results is not useful, as different instruments were applied for index assessment.

The present study showed that the prevalence of problematic Internet use in first-year was higher than in second-year students. The second-year course is probably more complicated than the first-year course, and students have less free time to surf the Internet. Also, the present study results showed that problematic Internet use (PIU) was significantly higher in younger students, similar to other studies [29–31]. This result might be due to increased students' knowledge and awareness about the Internet's proper use. Meanwhile, the problematic Internet use in married students was less than in single ones, which was statistically significant. Nasiri et al. and Ozgur et al. reported the same results [30, 32]. It can be due to single students having fewer tasks than married ones and they also have more free time.

Moreover, according to the present study results, the mean OHIP score was significantly higher in males. In other words, the oral health-related quality of life in male students was poorer than female students. In contrast, another study has shown that there was no difference between male and female dentistry students in the mean OHIP score [33]. Drachev et al. have reported that female medical and dental student’s oral hygiene was poorer than male students [34]. These differences might be due to cultural variances.

Also, in the present study, it was observed that the mean OHIP score was positively and directly related to age. In other words, oral health-related quality of life was poorer in senior students, similar to Drachev et al.’s study [34]. Furthermore, the oral health-related quality of life in second-year students was better than in first-year students. Nevertheless, it was not statistically significant. On the other hand, Kawamura et al. and Rong et al. showed that oral health-related quality was poorer in higher semester students [35, 36].

There are some limitations to the present study. First, the cross-sectional design does not allow us to draw conclusions about a causal relationship between problematic Internet use and oral health-related to quality of life. Therefore, longitudinal research is needed in the future to confirm their temporal relationships. Second, our samples were derived from only one university of medical sciences; so, caution needs to be exercised when attempting to generalize the present findings to the entire Iranian medical and dental students in basic sciences education period. Future research should examine whether the present study results could be replicated in other medical sciences universities in our country. Third, since the current study relied on self-report questionnaires, there are common issues that self-administered surveys would encounter, such as potential reporting bias. However, this survey was anonymous and employed empirically validated measures for our main study variables.

## Conclusion

The present study showed that problematic Internet use was significantly associated with oral health-related quality of life (OHRQoL) among first and second-year medical and dental students. Thus, the students with problematic Internet use experienced a poorer oral health-related quality of life than average Internet users. Furthermore, appropriate preventive and interventional strategies need to be developed to encourage rational use of the Internet to protect the users' oral health, especially among medical and dental students.

## Data Availability

The datasets used and/or analysed during the current study are available from the corresponding author on reasonable request.

## Abbreviations

PIU: Problematic Internet Use
OHRQoL: Oral Health-Related to Quality of Life
OHIP: Oral Health Impact Profile
WHO: World Health Organization
QoL: Quality of Life
